# Quantitative impact of Dixon μmap variability in dual-time-point brain PET/MR

**DOI:** 10.1186/2197-7364-2-S1-A74

**Published:** 2015-05-18

**Authors:** Kimberly Jackson, Rachel Bartlett, Kent Friedman, Timothy Shepherd, Thomas Koesters, Jose Teruel, Mathias Fenchel, Gerardo Hermosillova-Valadez, David Faul, Fernando Boada

**Affiliations:** Center for Biomedical Imaging, Department of Radiology, NYU Langone Medical Center, New York, USA; Siemens Healthcare, USA

Dixon-based MR is acquired for attenuation correction of brain PET during PET/MR. Early adopters of PET/MR have noted variability in the performance of Dixon-based tissue segmentation, and questions exist regarding potential impacts on quantitative accuracy in dual-time-point studies. Ten patients injected with 10 mCi FDG underwent dual-time-point clinical brain PET/MR on a Siemens mMR, with image reconstructions based on data acquired at 45 – 60 and 75 – 90 minutes. Dixon μmaps were obtained the time of FDG injection and at 75 minutes. 8 cc of Gadavist was injected for post-contrast MR at 30 minutes. Subjects were removed from the table and re-positioned prior to the 75 minute scan. The delayed μmap was registered to the original μmap using MIMneuro. The aligned image data was copied onto the original μmap DICOM file using Matlab. Early time-point PET data was reconstructed using both the early and delayed μmaps. Atlas-based segmentation was performed to compare regional SUV values. When comparing the delayed versus original μmap reconstructions, regional SUV values varied on average by +1.9% for both SUVmax and SUVmean. For large brain structures, SUVmax and SUVmean varied by -0.5% to +5.6% and -0.2 to +4.7%, respectively. For deep brain structures, SUVmax using the delayed reconstruction varied by -0.5% to +3.7% and SUVmean varied by -0.3% to +3.6%. Most differences in SUV were higher when using the delayed μmap. There is variability in regional SUV values for brain PET data reconstructed using two different aligned Dixon acquisitions performed on subjects undergoing repeat same-day PET/MR imaging, with SUV values ~2% higher using the delayed μmap. This variability may impact results in dual-time-point brain PET/MR.

